# Gastrocnemius recession: Discrepancies in the literature

**DOI:** 10.1016/j.jor.2024.04.005

**Published:** 2024-04-03

**Authors:** Nicholas Ehrenborg, Connor Davis, Jacob Tremoulis, Brett Bussert, Nicholas A. Cheney, Patrick O'Connor

**Affiliations:** a Ohio University Heritage College of Osteopathic Medicine, Athens, USA; b Foot and Ankle Surgery, OrthoNeuro, Columbus, USA; c Department of Biomedical Sciences, Heritage College of Osteopathic Medicine, Ohio University, Athens, USA

**Keywords:** Gastrocnemius, Gastrocnemius-soleus, Recession, Equinus, Silfverskiold

## Abstract

A gastrocnemius contracture is a common problem that results in decreased ankle dorsiflexion that contributes to an array of foot and ankle ailments. A common surgical treatment for this condition is a gastrocnemius recession (GR). Many adaptations of the original procedure have been described. Misinterpretations of proper GR procedures have potentially caused confusion when selecting a treatment. This paper proposes to identify errors between the use of GR and gastrocnemius-soleus recession (GSR) procedure techniques in the current literature.

A systematic literature review was performed in June 2021, using the PubMed database and select orthopedic texts. Only studies that met the established criteria and either correctly or incorrectly described a GR or GSR procedure were included. After applying exclusion criteria, 108 publications were included. These articles and texts were reviewed for surgical technique and terminology errors in accordance with established parameters. The articles were classified as either: “Correct” or “Incorrect.”

Of the 108 publications and texts included, 18 articles incorrectly described either a GR or a GSR (16.67%). Ninety articles correctly described either a GR or a GSR (83.33%).

The literature supports the use of a GR to treat a gastrocnemius contracture. Inaccurate articles create confusion as to what exactly a GR entails. Sources of ambiguity included terminology, inconsistent anatomical zone definition, and technique selection. Due to this confusion, it is suspected that patient outcomes can be impacted. Postoperative outcomes of GSR patients are worse than GR patients. Further investigation is necessary to determine if performing the incorrect procedure negatively affects patient outcomes.

## 1 Introduction

The primary plantar flexor muscle group of the leg is the triceps surae. This is composed of the superficial, but powerful, gastrocnemius muscle and the deeper soleus muscle. The gastrocnemius originates from the medial and lateral femoral condyles and inserts onto the posterior calcaneus via the Achilles tendon. The soleus, conversely, originates from the fibula and tibia to insert onto the posterior calcaneus via the Achilles tendon. The primary function of the gastrocnemius is flexion at the knee and plantarflexion at the ankle due to crossing both joints. The primary function of the soleus includes plantarflexion of the ankle and maintaining sufficient standing posture throughout the gait cycle.^1^

Certain factors are known to contribute to a higher likelihood of gastrocnemius equinus contracture development. These include older age, wearing high-heeled shoes, immobility, and medical comorbidities.^2^ Given that two separate muscles can cause an equinus contracture, it is important to differentiate the specific contracted muscle. In 1924, Nils Silfverskiöld developed an orthopedic test that allowed the differentiation of a gastrocnemius-soleus contracture from an isolated gastrocnemius contracture. This was conducted via a knee flexion test. Silfverskiöld wrote, “if the equinus is due mainly to gastrocnemius contracture, it can be readily overcome by flexing the knee and relaxing the gastrocnemius. If this is not the case, the deformity is due to contracture of the soleus, long toe flexors, tibialis posterior or peroneal [longus or brevis] muscles, to contracture of the posterior capsule of the ankle joint or a bony deformity such as an anterior bony block or a deformation of the body of the talus”.^3^ By ensuring a proper Silfverskiöld test technique is performed in clinical practice, one can accurately distinguish between the two major plantar flexor muscles of the lower leg as the cause of an equinus contracture.^4^

When standing, the foot is meant to function as a tripod with the calcaneus serving as one point, and the first metatarsophalangeal joint and forefoot serving as the two other points.^1^ Lezak^5^ noted that “the first metatarsal bears about 30–50% of the [foot's] weight.” If an individual has a pathologically shortened gastrocnemius (gastrocnemius equinus contracture), the heel is elevated which transfers an abnormal load to the front of the foot.^6^ Increased stress and irritation placed upon the posterior and anterior tibial tendons develops into various foot and ankle pathologies because of an imbalance in weight distribution.^7^ The anterior tibial tendon, acting as the primary dorsiflexor of the foot, can develop tendonitis when antagonizing a contracted gastrocnemius. The posterior tibial tendon is responsible for an integral role in foot biomechanics as it stabilizes the medial longitudinal arch of the foot.^8^ Excess stretch, inflammation, and irritation of the posterior tibial tendon, due to a pathologically shortened gastrocnemius, can cause its weakening and lengthening.

Habbu et al. developed a classification system for the “development of certain foot deformities [and arch collapse] resulting from compensation due to an equinus contracture”.^9^ This system classifies foot pathologies into five distinct categories based on whether the pain or deformity is present in the soft tissues, forefoot, midfoot, hindfoot, or ankle. Some deformities include, but are not limited to, plantar fasciitis, metatarsalgia, hypermobile first ray, Achilles pain, hypermobile first ray, hallux valgus, lesser toe deformity, metatarsal stress fracture, midfood arthritis, hindfoot valgus, peritalar subluxation, and ankle arthritis/deltoid ligament failure.^9^ With numerous pathologies resulting from a gastrocnemius contracture, accurate diagnosis is crucial. First-line treatments include rest, orthotics, stretching, and physical therapy.^9^ Upon failure of conservative treatment options, a subsequent recession of the pathologically contracted muscle is surgically indicated to prevent further strain on the arch.

**Fig. 1:**
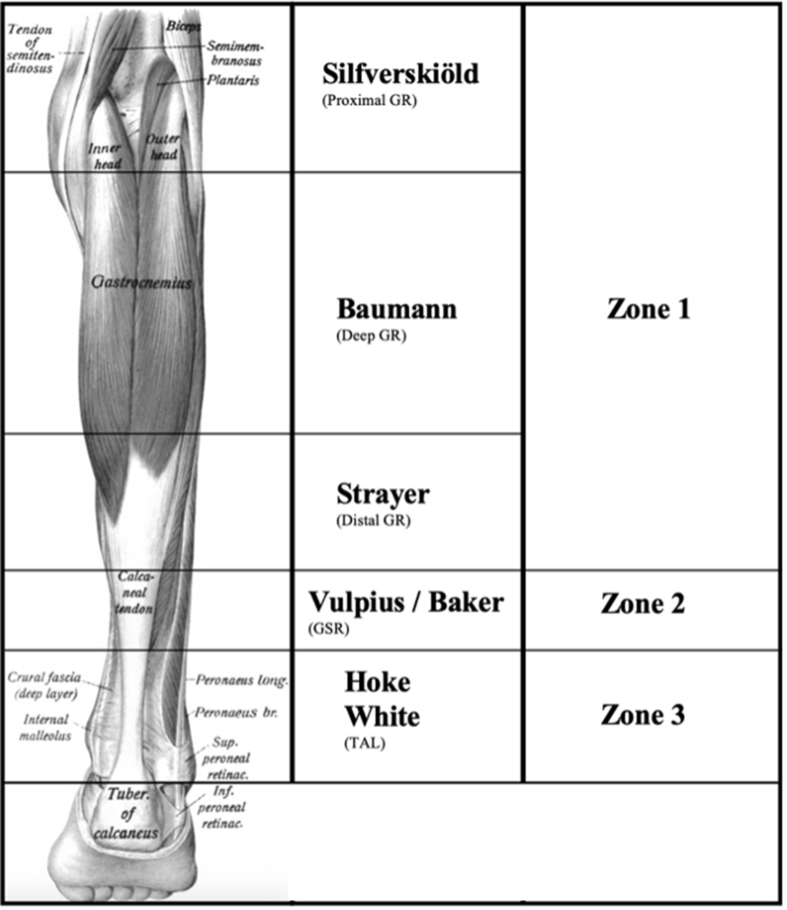
Demonstrates the three anatomic zones of the triceps surae and in which zone each recession procedure is performed. Image obtained and altered from Wikimedia Commons, a public domain resource. Original image author was Dr. Johannes Sobotta and was published in Sobotta's Atlas and Text-book of Human Anatomy 1909. Artwork is in the public domain in the United States because it was published before January 1, 1927.

Once an equinus contracture has been diagnosed as either a gastrocnemius-soleus contracture or an isolated gastrocnemius contracture and has failed conservative treatment, surgical options are indicated. When a patient has a gastrocnemius-soleus contracture, their Silfverskiöld test is positive with the knee both extended and flexed. This is indicative of the triceps surae muscle group being contracted. To treat this, a gastrocnemius-soleus recession (GSR), or more distal Tendo-Achilles lengthening (TAL) is indicated. When a patient has an isolated gastrocnemius contracture, their Silfverskiöld test is positive with the knee extended, but negative with the knee flexed. To treat this, a gastrocnemius recession is indicated. Hsu et al. reports the recession techniques as described by the original authors and shown in Fig. 1^10^. The gastrocnemius recession (GR) techniques include:

a) Silfverskiold: Proximal Medial and Lateral Gastrocnemius Release
b) Barouk: Isolated Proximal Medial Gastrocnemius Release
c) Baumann: Midmuscle Gastrocnemius Recession in the medial head of the Gastrocnemius
d) Strayer: Distal Gastrocnemius Recession

Hsu also reports the Gastrocnemius-Soleus recession techniques as initially described, which include:

e) Vulpius: Gastrocnemius-Soleus Recession (GSR)
f) Baker: Gastrocnemius-Soleus Recession (GSR)

Finally, Tendo-Achilles Lengthening procedure techniques are reported by Carpenter et al.^11^ and Chen et al.^12^ which include:

g) White: Double Hemisection Tendo-Achilles Lengthening
h) Hoke: Triple Hemisection Tendo-Achilles Lengthening

The primary difference in the 3 types of procedures described is the location where the lengthening or recession of the triceps surae muscle complex occurs. Tinney et al.^13^ provides a three-zone description of the triceps surae muscle group, shown in Fig. 1. “Zone-1 is from the origin of the proximal gastrocnemius to the most distal fibers of the medial gastrocnemius belly. Zone-2 is from the termination of the medial gastrocnemius belly to the distal extent of the soleus muscle fibers. Anatomically, zone-2 consists of the conjoined tendon of the gastrocnemius aponeurosis and the soleal fascia. Zone-3 consists of the tendo-Achilles, distal to the soleal muscle fibers.”

Zone-2 consists of two separate sub-zones: one before the conjoining point of gastrocnemius and soleus and one of a completely conjoined tendon of the gastrocnemius aponeurosis and the soleal fascia.^13^ Because there are two anatomical differences within one zone and no two patients share the exact anatomy, the potential for confusion is present. Additionally, the absence of a universal standard to systematically demarcate one anatomical zone from another presents further basis for confusion.^14^ The Strayer procedure and the Vulpius procedure are performed in adjacent anatomical zones, separated by millimeters.^15^ Differentiation between zones is crucial due to the minimal difference between resecting just the gastrocnemius muscle and resecting the entire triceps surae muscle complex. This small difference creates larger biomechanical changes in the functionality of the plantar flexor muscles postoperatively.^16^, ^17^, ^18^ More importantly, GSR/TAL have worse postoperative outcomes than isolated GR.^19^ Due to these varying clinical outcomes, the importance of performing the correct procedure in the correct zone is significant. This paper analyzes how GR is presented in the relevant literature, to clarify the difference between a GR and a GSR. To the authors’ knowledge, this is the first systematic review in the English language to investigate the number of articles correctly describing or differentiating a GR from a GSR/TAL.

## 2 Methods

A literature review was performed in June 2021, by the investigators, using the PubMed database with the following search terms: “((((((((((gastrocnemius OR gastrocsoleus) AND (recession OR release OR lengthening)) OR (Gastrocnemius equinus)) OR (Gastrocnemius contracture)) OR (Silfverskiöld)) OR (Baumann (gastrocnemius))) OR (Strayer)) OR (Vulpius)) OR (Baker (gastrocnemius))) NOT (Pediatric)) NOT (simulation)”

Only studies that correctly or incorrectly described a gastrocnemius or gastrocnemius-soleus recession/tendo-Achilles lengthening procedure were included for review by these authors. For the purpose of this study, a GR was defined as release of the gastrocnemius muscle or tendon without release of soleus fascia, muscle, or tendon. GSR/TAL was described as release of both the gastrocnemius and soleus muscle or tendon. Only studies that met these criteria were included. This search yielded 25,608 publications. Exclusion criteria included articles that matched keywords but did not include a description of the procedure, articles that focused on pediatric patients, computer simulations, and basic science articles. After applying exclusion criteria, 108 publications remained and were included in this literature review.

These 108 articles and texts were then reviewed for surgical technique and terminology errors. The articles were classified in one of two ways: “Correct” or “Incorrect.” Collected data were compiled in an Excel spreadsheet and subsequently analyzed.

## 3 Results

One hundred and eight articles and textbook references met inclusion criteria and were analyzed. Upon analysis, eighteen articles were deemed incorrect. Twenty-two articles described endoscopic GRs, with 4 of them incorrectly describing the procedure.

## 4 Discussion

GRs and GSRs/TALs are common procedures to address a multitude of foot and ankle pathologies. With low complication rates, low infection rates, and high correction rates, these procedures are performed frequently in the foot and ankle community. With the literature review resulting in 108 articles that met inclusion criteria, it is evident that this is a well-studied topic. The first documented GSR occurred in 1913. There have since been many variations of this original procedure. Some of these were intentional, while other changes have occurred due to a language barrier and the significant time since publication. This project has determined that error is prevalent in the literature, regarding proper surgical technique and specificity of language when describing the performed procedure.

Table 1 shows that the error rate of articles confusing a GR and a GSR is as high as 16.67%. These incorrect articles included but were not limited to, calling a Vulpius procedure a GR, stating that a GR included recession of both gastrocnemius and soleus, and including GR techniques when listing GSR techniques.

**Table 1:** Display of data obtained from the literature review. 108 articles were analyzed.

Correct Articles	90	83.33%
Incorrect	18	16.67%
Incorrect Due to Vulpius Error	8	44.44%
Incorrect + Endoscopic	36	33.34%

The Vulpius procedure, as reported by Hsu et al. who analyzed the original German publication, is a transverse recession of both the gastrocnemius and the soleus muscle tendons.^10^ This means that Vulpius is a GSR technique and should not be used to treat an isolated gastrocnemius contracture. Some individuals incorrectly believe that the Vulpius technique is the shape of the incision (inverted V shape).^19^ The Vulpius technique, however, is differentiated from other techniques in that it cuts both the soleus and gastrocnemius. The location of the cut is the distinguishing factor in the Vulpius technique, not the shape of the cut (inverted V vs. transverse).^13^ This distinction serves as one of the primary areas of confusion surrounding this technique. Of 16.67% of articles that had an incorrect description of GR, 44.44% were due to confusion regarding the Vulpius procedure.

One potential cause of this confusion could be the inconsistency with which the phrase “conjoined tendon” is used in the literature. When using the phrase “conjoined tendon/musculotendinous junction,” some authors were referring to the combination of the gastrocnemius tendon and that of the soleus muscle.^15^^,^^20^, ^21^, ^22^ This leads into and forms the Achilles tendon. Other authors, however, used the phrase “conjoined tendon/musculotendinous junction” to describe the combination of the medial and lateral gastrocnemius heads into one tendon.^10^^,^^16^^,^^18^^,^^23^^,^^24^ Repeatedly, articles and textbooks describe making an incision just distal to the “conjoined tendon.” Clarification is required regarding which “conjoined tendon” is necessary for selecting the proper surgical procedure. An isolated gastrocnemius recession would refer to the former definition of conjoined tendon, that of the two heads of gastrocnemius. A GSR, however, requires incision of the soleus and gastrocnemius tendons together. The latter recession matches the first definition.

Whereas both the gastrocnemius and soleus muscles serve a similar role at the ankle joint, their slight anatomical and functional differences underscore why a distinction between recession procedures is crucial. These differences include soleus' greater role in ankle plantarflexion, and in particular, during prolonged walking, and standing. Additionally, gastrocnemius’ primary role is knee and ankle plantarflexion along with “pushing-off” when completing stance phase and initiating a swing phase of the stride cycle.^20^ Without proper classification and understanding of which recession is being performed, the potential for further complications is appreciable if surgeons incorrectly perform a GSR for an isolated gastrocnemius contracture.

Table 1 reports the significant confusion in the literature regarding GR, GSR, and TAL procedures. This confusion leads to the potential for incorrect procedures being performed. These incorrect procedures have resulted in varying patient outcomes. Multiple evidence-based studies have shown that patients who undergo gastrocnemius recessions exhibit higher postoperative plantarflexion strength and improved angles of dorsiflexion than patients following GSR/TAL procedures.^17^^,^^19^^,^^25^^,^^26^ Thus, if a patient receives a GSR or TAL for an isolated gastrocnemius contracture, they will likely develop more functional deficits than necessary.^27^ Consequently, improper education, diagnosis, and procedure selection for equinus contracture can lead to worse outcomes for patients.

The simplest way to avoid negative outcomes from an unnecessary procedure is by the correct initial diagnosis. This is done via the Silfverskiöld test, as described above. Silfverskiöld's test, when performed correctly, informs the physician of which muscle(s) is contracted. Silfverskiöld's test, when performed inaccurately, such as only performing the test with one hand, could lead to inadequate diagnoses of foot and ankle pathologies.^9^ If a patient achieves greater foot dorsiflexion with the knee flexed than with the knee extended, the patient has an isolated gastrocnemius contracture. If a patient achieves no dorsiflexion regardless of knee position, the patient has a contracted triceps surae. This straightforward test quickly and accurately provides a diagnosis and eliminates the need for an intraoperative range of motion test to determine if more recession is necessary.

Another action to prevent negative results from an unnecessary procedure is recession in the proper anatomical zone. Whereas no universal standard is present, there are widely adopted demarcations that exist in the posterior lower leg 15,22. The distal aspect of zone one is where a surgeon would perform a Strayer procedure for an isolated gastrocnemius contracture. The proximal aspect of zone two is where a surgeon would perform a Vulpius/Baker procedure for a contracted triceps surae. If particular attention is paid to where the combination of the gastrocnemius tendon joins with the soleus tendon, then there should be no confusion as to which procedure a patient received. Additionally, endoscopic GRs present another challenge in ensuring the recession is performed in the proper zone. Depending on the location of the portal created and the depth of the cutting blade and force at which the tissue is released it is conceivable that the soleus fascia/muscle can be released accidently with these endoscopic procedures. Open recessions allow the surgeon to palpate the end of zone one and beginning of zone two, where the muscles’ tendons conjoin. This palpation is not possible with endoscopic GRs. This study classified endoscopic GR articles as “correct” so long as they did not describe a GSR and call it a GR. However, given that the surgeon cannot palpate the musculotendinous junction of the gastrocnemius and the soleus, one could argue that endoscopic GRs are not always only cutting the gastrocnemius aponeurosis, and the soleus could be recessed as well. If this study included endoscopic GRs in the “incorrect” category of Table 1, the percentage of articles that meet the definition of incorrect, rises from 16.67% to 33.34%. Current literature suggests that endoscopic GRs result in fewer complications than open GRs.^28^, ^29^, ^30^ However, a surgeon must decide if the benefit of performing an endoscopic recession is worth the potential risk of cutting the soleus unnecessarily.

Some results were surprising. One very common orthopedic textbook had the Vulpius procedure incorrectly listed as a GR, not as a GSR. The inconsistency in the foot and ankle community on the language used regarding the use of “conjoined tendon/musculotendinous junction” to describe different areas in the gastroc-soleus complex was extremely prevalent. Finally, the lack of a universal standard regarding anatomical zone demarcation of the lower leg was noted.

This study is not without its limitations. First, only studies published in the English language were included. Potentially high-quality articles were also excluded due to infrequent full-text access restrictions through this academic center. Another potential limitation is selection bias, which was mitigated by utilizing inclusive search parameters, multiple sources, and three researchers selecting articles for inclusion. Any ambiguous articles were discussed amongst the team and categorized unanimously.

Future steps are needed to continually improve patient care and clinical outcomes related to this topic using evidence-based findings. Surgeons should reevaluate whether they are inaccurately performing a GSR/TAL procedure when treating an isolated gastrocnemius contracture. Surgeons should also reevaluate what procedures they should be performing based on proper Silfverskiöld utilization and results. Adoption of a universal standard for anatomical zone demarcation and procedural terminology is strongly recommended to minimize confusion. Finally, a study analyzing if the benefits of endoscopic GR outweigh the potential complications that arise from incidentally performing a GSR when that was not the intended procedure.

## 5 Conclusions

A review of the literature currently available for gastrocnemius recessions, gastrocnemius-soleus recessions, and tendo-Achilles lengthening procedures have identified an absence of a universal consensus regarding anatomical zone demarcation of the lower leg, accurate descriptive terminology, and proper procedural technique. As such, this situation can potentially lead to improper procedures being performed on patients, resulting in worse functional outcomes due to the important anatomical and functional differences between gastrocnemius and soleus. Determining an accurate initial diagnosis by properly performing the Silfverskiöld test preoperatively is crucial for selecting the appropriate recession procedure for each patient. This article serves to call attention to these issues, with the goal being education of the problem alongside the utilization of evidence-based medicine to provide optimum treatment for patients.

## Guardian

No guardian/parent consent.

## Ethical approval

Ethical approval was not sought for the present study because this study did not involve human subjects.

## Funding

This research did not receive any specific grant from funding agencies in the public, commercial, or not-for-profit sectors.

## CRediT authorship contribution statement

**Nicholas Ehrenborg:** Conceptualization, Methodology, Investigation, Writing – original draft, Reviewing, Editing. **Connor Davis:** Conceptualization, Methodology, Investigation, Writing – original draft, Reviewing, Editing. **Jacob Tremoulis:** Conceptualization, Methodology, Investigation, Writing – original draft, Reviewing, Editing. **Brett Bussert:** Investigation, Writing Final Draft, Reviewing, Editing, Visualization. **Nicholas A. Cheney:** Conceptualization, Methodology, Investigation, Writing – original draft, Reviewing, Editing, Validation, Supervision, Project administration. **Patrick O'Connor:** Project administration.

## Declaration of competing interest

None.
